# Supplementary material for: Probabilistic behavioral aggregation: A case study on the Nordic power grid
Source: PLoS One. 2025 Aug 25;20(8):e0322328. doi: 10.1371/journal.pone.0322328 (PMC12377621; doi:10.1371/journal.pone.0322328)
Supplement: S1 Appendix — (PDF) [file pone.0322328.s001.pdf]

Implementation

The key technical challenge in evaluating the probabilistic distance and solving the optimization problem is obtaining fully differentiable, realistic, and fast models of power grids. The following summarizes the computational methods and software used to address this complex joint-optimization problem.

The entire software stack is implemented using the Julia programming language. One of Julia's most significant features is its support for "differentiable programming," which enables the efficient and accurate computation of derivatives for arbitrary Julia programs. This capability facilitates using gradient-descent-based optimization methods in conjunction with differential equation solvers. Numerically, the minimization of the behavioral distance is performed using a gradient-descent approach with the ADAM optimizer.

Our power system simulations are built on two Julia software packages: `BlockSystems.jl` and `NetworkDynamics.jl`. Both packages are designed for highly efficient transient stability simulations of power grids. They allow users to design power systems that are modular and equation-based while maintaining high performance and detail.

The benchmark results between the system and specification were achieved with this simulation setup. We compare the times for the evaluation of one sample. We have employed `BenchmarkTools.jl` for the performance tracking. The benchmark has been performed on a Dell Inc. Latitude 7440 with a 13th Gen Intel i7-1365U (12) CPU.

Analytic Baseline

Following the simplifications given in Section 3.1 of the main paper, we derive the analytical baselines. The following system of swing equations describes the approximated system:

$$\dot{\boldsymbol{\theta}} = \boldsymbol{\omega} \tag{1}$$

$$2\boldsymbol{H}\dot{\boldsymbol{\omega}} = -\boldsymbol{D}\boldsymbol{\omega} + \boldsymbol{\Delta P} + \boldsymbol{u} \tag{2}$$

where $\boldsymbol{\Delta P} = \boldsymbol{P_{fix}} - \boldsymbol{P_e}$ and $\boldsymbol{u}$ is the control input. Multiplying Eq (2) by the unity vector $\boldsymbol{1_n^T}$ results in the following relation:

$$\sum_i^M 2H_i\dot{\omega}_i = -\sum_i^M D_i\omega_i + \sum_i^M \Delta P_i + \sum_i^M u_i. \tag{3}$$

Where i is the bus index. We define the the total power mismatch ΔP_{total} as:

$$\Delta P_{total} = \Delta P_i. \tag{4}$$

Starting again from Eq (3), we assume that the asymptotic state is fully synchronized, meaning that $\omega_i = \omega^*$, but not necessarily synchronized at the operating frequency ω_0, which results in the following equation:

$$0 = \Delta P_{total} - \omega^* \sum_i^M D_i + \sum_i^M u_i^*. \tag{5}$$

For the P-controller, there is no additional control, i.e., $u = 0$, which results in the following equation for the asymptotic frequency ω^*:

$$\omega^* = \frac{\Delta P_{total}}{\sum_i^M D_i}, \tag{6}$$

where M is the total number of buses, as the total power mismatch ΔP_{total} is the same for system and specification, we find $D_{base} = \sum_i^M D_{i,sys} = MD_{sys}$ for the baseline.

For the I-controller, the asymptotic control action u_i^* becomes:

$$u_i^* = -K_i \int_0^\infty \omega_i(t) \tag{7}$$

For the PI controller, it is known that the asymptotic error, in our case the asymptotic frequency, always reaches zero. Using these result in Eq (5) results in the following relation for the asymptotic frequency ω^*:

$$0 = \Delta P_{total} - \omega^* \sum_i^M D_i - \sum_i^M K_i \int_0^\infty \omega_i(t) \tag{8}$$

$$\omega^* = 0 = \frac{\Delta P_{total} - \sum_i^M K_i \int_0^\infty \omega_i(t)}{\sum_i^M D_i}. \tag{9}$$

thus the baseline integral gain becomes $K_{base} = MK_{sys}$.

For the leaky integral control, we use the separation of variables to solve the first-order differential equation. To find the asymptotic control action u^*, we again use the simplification that in the asymptotic state $\omega_i = \omega^*$:

$$\int \frac{dt}{T_i} = \int \frac{dy_i}{\omega_i - G_i \cdot y_i} \tag{10}$$

$$u_i = -\frac{1}{G_i}(e^{-t(G_i/T_i)} - \omega) \tag{11}$$

$$u_i^* = \frac{\omega^*}{G_i}. \tag{12}$$

As e^{-tG_i/T_i} goes to zero in the limit of $t \to \infty$, we can not define a baseline for T_i as it does not influence the asymptotic state nor the initial response of the system. Using the leaky asymptotic control gain and Eq (5), we find the asymptotic frequency for the system with leaky integral controllers:

$$0 = \Delta P_{total} - \omega^* \sum_i^M D_i + \omega^* \sum_i^M \frac{1}{G_i} \tag{13}$$

$$\omega^* = \frac{\Delta P_{total}}{\sum_i^M D_i - \sum_i^M (1/G_i)}. \tag{14}$$

Meaning that we should choose $1/G_{base} = \sum_i^M (1/G_{i,sys}) = M/G_{sys}$ such that the N5 system and specification end up in the same asymptotic state.
