# Supplementary figures and images for: Probabilistic behavioral aggregation: A case study on the Nordic power grid
Source: PLoS One. 2025 Aug 25;20(8):e0322328. doi: 10.1371/journal.pone.0322328 (PMC12377621; doi:10.1371/journal.pone.0322328)

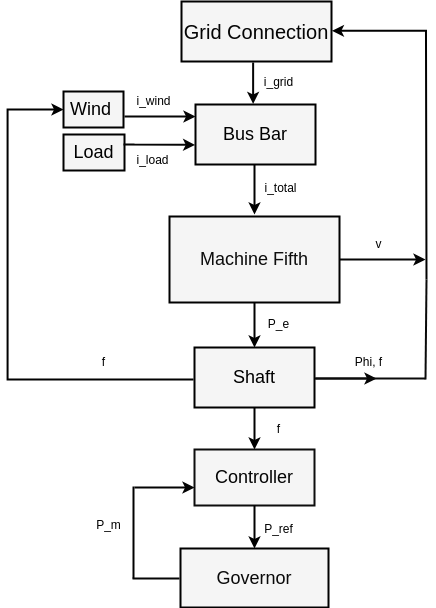

Supplement: S1 Fig — (PNG) [file pone.0322328.s002.png]

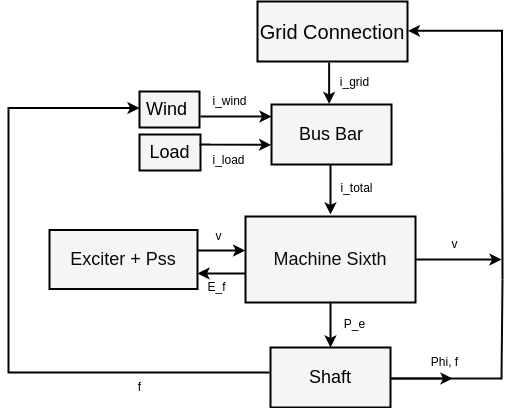

Supplement: S2 Fig — (PNG) [file pone.0322328.s003.png]

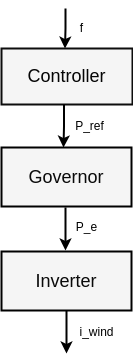

Supplement: S3 Fig — (PNG) [file pone.0322328.s004.png]
