# Supplementary material for: Probabilistic behavioral aggregation: A case study on the Nordic power grid
Source: PLoS One. 2025 Aug 25;20(8):e0322328. doi: 10.1371/journal.pone.0322328 (PMC12377621; doi:10.1371/journal.pone.0322328)
Supplement: S1 Table — (PDF) [file pone.0322328.s005.pdf]

	P	PI	PLI
System	18.87 ms $\pm$ 7.05 ms	19.25 ms $\pm$ 6.89 ms	20.10 ms $\pm$ 5.66 ms
Spec	2.94 ms $\pm$ 2.88 ms	2.82 ms $\pm$ 2.75 ms	3.02 ms $\pm$ 2.81 ms
Speed-up	$\approx$ 6.42	$\approx$ 6.83	$\approx$ 6.65

Comparison of the simulation times for the system and specification using random mode fluctuations.
