# Supplementary material for: Probabilistic behavioral aggregation: A case study on the Nordic power grid
Source: PLoS One. 2025 Aug 25;20(8):e0322328. doi: 10.1371/journal.pone.0322328 (PMC12377621; doi:10.1371/journal.pone.0322328)
Supplement: S2 Table — (PDF) [file pone.0322328.s006.pdf]

	P	PI	PLI
System	141.03 ms ± 6.93 ms	144.36 ms ± 10.76 ms	193.46 ms ± 6.47 ms
Spec	6.66 ms ± 5.24 ms	6.38 ms ± 5.16 ms	10.35 ms ± 5.17 ms
Speed-up	≈ 21.18	≈ 22.62	≈ 18.69

Comparison of the simulation times for the system and specification using the realistic demand fluctuations.
