# Supplementary material for: Probabilistic behavioral aggregation: A case study on the Nordic power grid
Source: PLoS One. 2025 Aug 25;20(8):e0322328. doi: 10.1371/journal.pone.0322328 (PMC12377621; doi:10.1371/journal.pone.0322328)
Supplement: S3 Table — (PDF) [file pone.0322328.s007.pdf]

	P	PI	PLI
Baseline o_{base}	3.425 ± 0.643	3.688 ± 0.664	3.653 ± 0.686
Initial d^{ρ}_{init}	2.138 ± 0.39	1.08 ± 0.194	2.272 ± 0.41
Tuned d^{ρ}_{end}	0.028 ± 0.006	0.067 ± 0.016	0.043 ± 0.009
Resampled d^{ρ}_{init}	0.025 ± 0.009	0.068 ± 0.025	0.042 ± 0.01

Behavioral distance d^{ρ} between system and specification at the different steps in the tuning pipeline. The error is given by the standard deviation.
