# Supplementary material for: Probabilistic behavioral aggregation: A case study on the Nordic power grid
Source: PLoS One. 2025 Aug 25;20(8):e0322328. doi: 10.1371/journal.pone.0322328 (PMC12377621; doi:10.1371/journal.pone.0322328)
Supplement: S4 Table — (PDF) [file pone.0322328.s008.pdf]

	P	PI	PLI
Baseline o_{base}	0.21 ± 0.09	0.522 ± 0.249	0.423 ± 0.242
Initial d^{ρ}_{init}	0.173 ± 0.095	0.187 ± 0.092	0.205 ± 0.112
Tuned d^{ρ}_{end}	0.002 ± 0.001	0.008 ± 0.004	0.019 ± 0.008
Resampled d^{ρ}_{init}	0.003 ± 0.001	0.006 ± 0.002	0.011 ± 0.004

Behavioral distance d^{ρ} between system and specification at the different steps in the tuning pipeline. The error is given by the standard deviation.
